# First person – Emily Jones, Zoe Matthews and Lejla Gul

**DOI:** 10.1242/dmm.039511

**Published:** 2019-03-18

## Abstract

First Person is a series of interviews with the first authors of a selection of papers published in Disease Models & Mechanisms (DMM), helping early-career researchers promote themselves alongside their papers. Emily Jones, Zoe Matthews and Lejla Gul are co-first authors on ‘Integrative analysis of Paneth cell proteomic and transcriptomic data from intestinal organoids reveals functional processes dependent on autophagy’, published in DMM. Emily is a postdoctoral research scientist in the lab of Professor Simon Carding at Quadram Institute, Norwich, UK, investigating host-microbe interactions at the intestinal epithelial barrier. Zoe is a medical student (research completed during PhD) in the lab of Prof. Tom Wileman at Biomedical Research Centre, University of East Anglia, Norwich, UK, investigating stem cell biology, focussing on the small intestine. Lejla is a PhD student in the lab of Dr Tamas Korcsmaros at Earlham Institute, Norwich, UK, investigating the effects of environmental (e.g. microbes) and genetic factors on the human autophagy process.


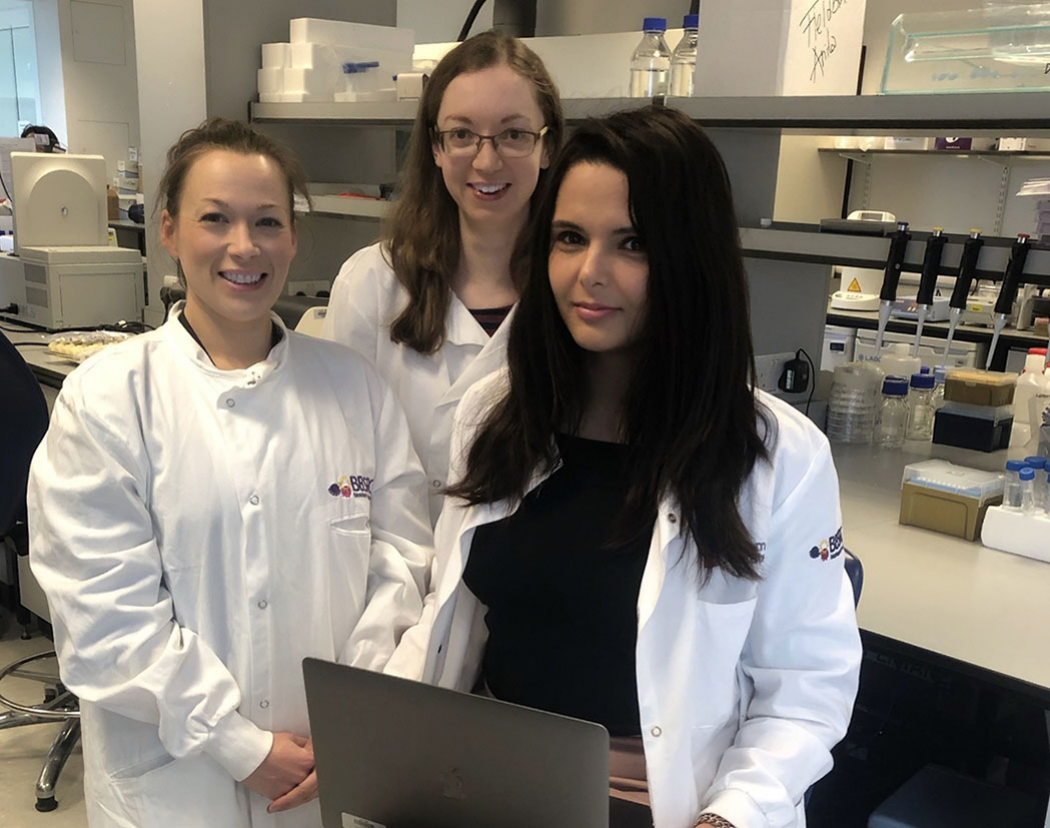


**Emily Jones, Zoe Matthews and Lejla Gul**

**How would you explain the main findings of your paper to non-scientific family and friends?**

EJ & ZM: Our work combined experimental biology and computational analysis. We started out in a lab, growing mini-guts (3D organoid cell cultures that mimic the intestine). We had two types of organoids: those that acted like a normal healthy small intestine and those that had an abnormality in a recycling protein (lack of the *Atg16l1* gene). This deficient model is relevant to study Crohn's disease (CD), which is an inflammatory condition of the gastrointestinal tract, as patients suffering from CD have an altered form of this gene. We aimed to investigate the effect of *Atg16l1* knockout on cellular functions using an organoid culture model.

LG: To prove our hypothesis, we developed a method combining both computational and experimental biology. We used this new pipeline to assess the effect of the *Atg16l1* knockout, which causes autophagy impairment. Autophagy is a cellular degradation process targeting organelles, pathogens and specific proteins. We predicted the effect of protein-level changes due to autophagy impairment on cellular functional processes. We measured the quantity of proteins showing different abundances in organoids lacking the *Atg16l1* gene, compared with wild types. Following data analysis, we identified functions that could have potentially been altered, and verified these experimentally.

**What are the potential implications of these results for your field of research?**

CD affects millions of people worldwide, but currently there is no cure for the disease. We have generated a Paneth-cell-enriched 3D organoid system to model the potential changes during the disease and discover the molecular background. We have implemented a bioinformatic pipeline to analyse systems-level changes in intestinal cells. As a result, potential functional changes related to CD were discovered, providing greater insight into the underlying mechanisms and aetiology of CD, and hopefully bringing research closer to an effective long-term treatment for patients.

“Potential functional changes related to CD were discovered, providing greater insight into the underlying mechanisms and aetiology of CD, and hopefully bringing research closer to an effective long-term treatment for patients.”

**What are the main advantages and drawbacks of the model system you have used as it relates to the disease you are investigating?**

Organoids grown from intestinal stem cells differentiate into all the cells of the intestinal epithelium, providing a useful laboratory model, both in terms of structure and function. Creating and using cell-type-specific enriched systems helps us to discover *in vivo* processes in an *in vitro* environment, thereby giving a nearly true insight into protein abundance changes in Paneth cells. The drawbacks of lineage-directing organoids into Paneth cells is that the final cultures are not pure – some stem cells remain, keeping the organoids alive. Also, here we have investigated a model system lacking the *Atg16l1* gene, while in CD patients the *ATG16L1* gene is in a mutated form. Therefore, the knockout model we used can be considered as an extreme model, where autophagy is impaired.

**Figure:**
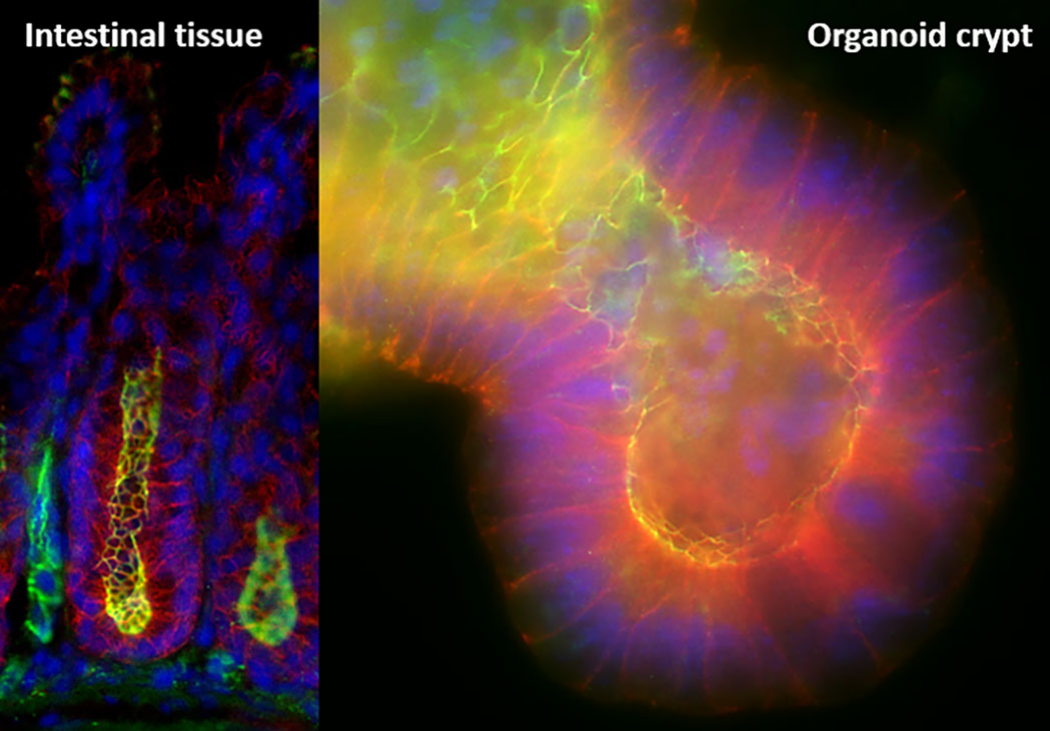
**Crypts of Lieberkühn from the mouse *in vitro* (left) and crypt from the *in vitro* organoid model (right).**

“A surprising outcome of the work was how many collaborations were formed in order to combine experimental biology with computational biology.”

**What has surprised you the most while conducting your research?**

EJ & ZM: A surprising outcome of the work was how many collaborations were formed in order to combine experimental biology with computational biology. A large volume of data was generated by many research groups and it was exciting to see the project progress from the original samples produced in the laboratory.

LG: Based on the literature, autophagy and apoptosis generally show a negative correlation under most homeostatic conditions but, surprisingly, we found a positive correlation between the two processes. We assume that, when autophagy is impaired, the downregulation of apoptosis could prevent the perturbed Paneth cells from sacrificing themselves, which would then be compensated for by outcomes such as upregulation of DNA damage repair functions, as suggested previously.

**Describe what you think is the most significant challenge impacting your research at this time and how will this be addressed over the next 10 years?**

Our findings are based on a murine cell culture, so the next steps will be translating our pipeline for human samples, then verifying the results in the human system. As this is developed, we hope that it will lead towards a more specific understanding of the *ATG16L1* mutation in CD, translating into clinical applications that provide more personalised, patient-specific treatment of CD. We are fortunate that Norwich Research Park continues to develop with the opening of the Quadram Institute, a state-of-the-art food and health research and endoscopy centre combining scientific excellence and clinical expertise, making this a more realistic goal over the next 10 years.

**What changes do you think could improve the professional lives of early-career scientists?**

EJ: I think it is important that early-career scientists are supported with longer-term projects that allow job security in order to enable them to publish their research and build their scientific career.

LG: The collaboration between different institutes and research groups is very important but one thing is more significant: collaboration among people from different areas of science. Early-career scientists should learn more about systems-level thinking using bioinformatics, outstepping the bounds of limited thinking.

**What's next for you?**

In the article, we focused mainly on the process of exocytosis when the protein turnover is disrupted in cells. Further experiments are needed to confirm the assumption about the role of DNA repair and apoptosis in Paneth cells and how the interruption of these processes could contribute to the pathogenesis of impaired autophagy-associated diseases such as CD. We look forward to testing our experimental-computational pipeline on patient-derived organoids.
